# A Case Report on Urethral Insertion of a USB Cable: Diagnosis and Management

**DOI:** 10.7759/cureus.78962

**Published:** 2025-02-13

**Authors:** Jonathan S Kim, Louis S Liou

**Affiliations:** 1 Urology, Drexel University College of Medicine, Philadelphia, USA; 2 Urology, Emerson Hospital, Concord, USA

**Keywords:** autoeroticism, endoscopic removal, minimally invasive surgery, urethral foreign body, usb cable

## Abstract

Self-insertion of foreign objects into the urethra for autoerotic or other motivations is uncommon yet potentially serious. We present a case of a urethral foreign body involving a thick USB cable, which was successfully removed using a semi-rigid ureteroscope after initial attempts with manual extraction and a rigid cystoscope were unsuccessful. This study highlights the importance of undertaking a tailored approach in the removal of more complex urethral foreign bodies and suggests a potential novel adaptation to an established procedural technique.

## Introduction

The self-insertion of foreign objects into the urethra and bladder is a rare but significant clinical challenge. Motivations for insertion include sexual stimulation, curiosity, psychiatric disorders, and intoxication [1,2]. Urgent intervention is of paramount importance; however, cases of foreign body insertions are commonly complicated by delayed presentation due to feelings of personal embarrassment [3,4]. Of those who do seek medical attention, the clinical presentation is varied, ranging from completely asymptomatic to lower urinary tract symptoms such as hematuria, dysuria, urinary frequency, and urinary retention [1-3]. Delayed diagnosis can lead to severe outcomes such as sepsis, urethral strictures, and long-term sexual dysfunction [2,4-6].

We describe a case of a male college student who inserted a USB cable into his urethra in the context of autoerotism, which required urgent urological intervention.

## Case presentation

A 21-year-old male college student presented with the insertion of a USB cable into his urethra. The patient reported a prior history of foreign body insertions for sexual stimulation, including cotton swabs and wire cables. However, this instance was the first in which he had difficulties with removal and required medical intervention.

**Figure 1:**
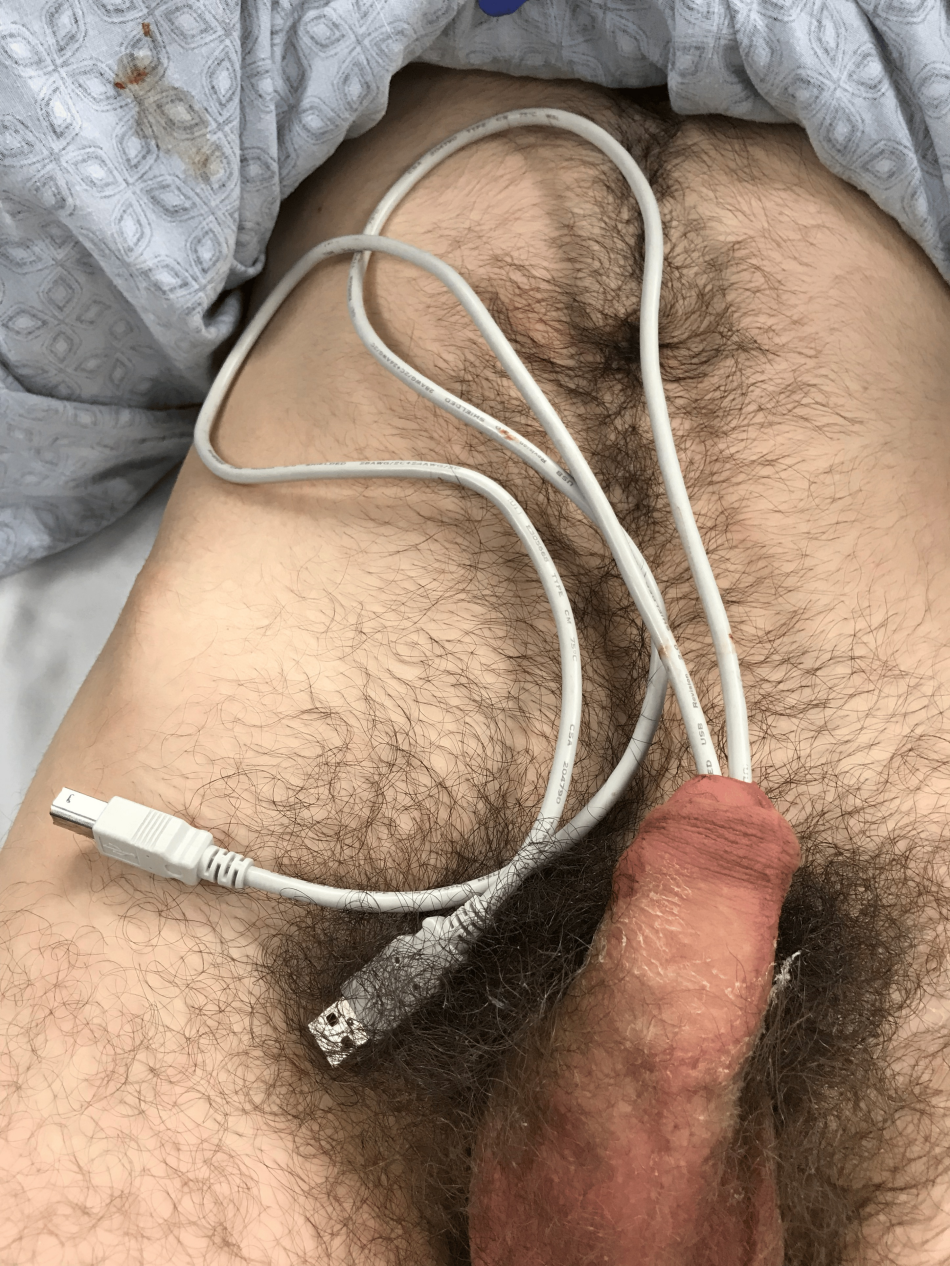
Urethral foreign body upon presentation to the emergency department, with both ends of the cable visible at the urethral meatus.

**Figure 2:**
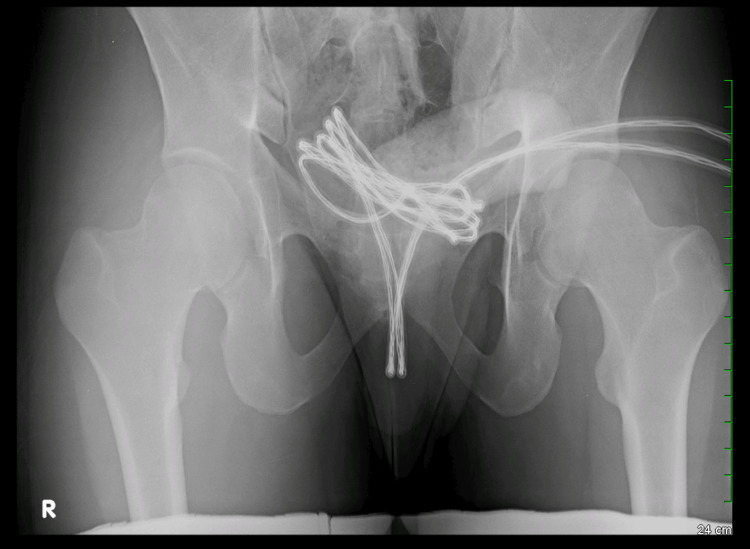
KUB radiograph depicting position of foreign body in urethra and bladder. KUB: kidney, ureter, and bladder

On admission, the patient was hemodynamically stable. Physical examination revealed a USB cable that appeared to be folded in half, with the distal ports of the wire protruding from the external urethral meatus and the middle portion within the urethra (Figure 1). A kidney, ureter, and bladder (KUB) radiograph revealed the exact position and shape of the cable extending into the bladder, which is presented in Figure 2. Attempts at manual extraction in the emergency department were unsuccessful due to resistance caused by the thick insulation of the cord and raised concerns for a potential knot or kink within the cable. The patient was counseled on the need for surgical intervention wherein an initial endoscopic approach would be attempted with escalation to an open procedure if necessary.

**Figure 3:**
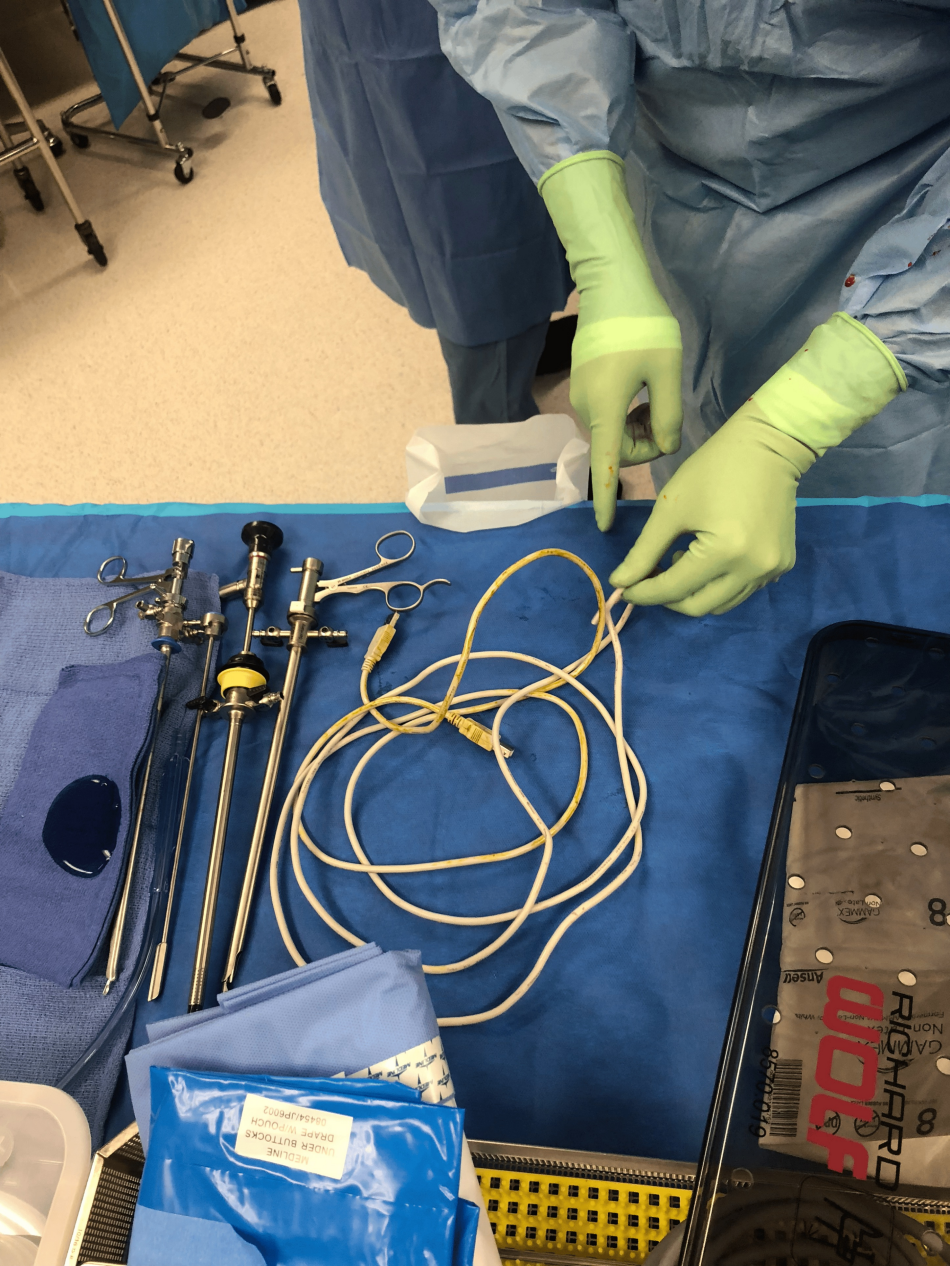
USB cable removed from the urethra.

In the operating room, the patient was placed under general anesthesia, and a semi-rigid ureteroscope was inserted alongside the USB cable, visualizing its trajectory into the bladder. Visual inspection revealed no knots but confirmed significant resistance. The cable was gently pulled under direct vision until its leading end was outside the urethra. Heavy scissors were used to cut the cable, facilitating the removal of the remaining cord segment (Figure 3).

Subsequent cystoscopy revealed minimal trauma to the urethra, and a Foley catheter was placed for a duration of one week. He was subsequently discharged with analgesics and oral antibiotics. A follow-up cystoscopy at one month revealed no evidence of strictures, and his recovery was uneventful. The patient did not return for further follow-up as he had graduated from college and relocated out of state.

## Discussion

The self-insertion of foreign bodies into the urethra, although rare, presents a significant clinical challenge due to the potential for severe complications and the varied motivations behind such actions. In the present case, the patient, a male college student, inserted a USB cable into his urethra for autoerotic purposes.

Common motivations that have been documented in the literature include sexual gratification, curiosity, and reckless behavior [1,3,7]. The association of urethral foreign bodies with mental health disorders is well-documented, with studies showing up to 35.6% of cases involving mood disorders, schizophrenia, or personality disorders [8].

Delayed presentation is a critical factor in worsening outcomes. Crawford et al. emphasize the importance of early imaging, including ultrasound, to prevent further migration and injury [9]. Diagnostic imaging, such as KUB radiographs or ultrasound, remains crucial for identifying the size, location, and nature of the foreign object.

The management of a urethral foreign body may be particularly challenging depending on the nature, number, and location of the retained object. Simple manual extraction can be an effective modality for smooth and mobile objects that protrude from the external urethral meatus [9]. For objects that are larger in size and more proximal in location, as in the present case, endoscopic techniques are preferred as a minimally invasive procedure which is associated with decreased disruption of the urethral anatomy and shorter hospitalization stays [9,10]. Furthermore, large or resistant objects may necessitate modifications or open surgical techniques, as demonstrated in cases involving complex or perforating foreign bodies [9,11].

The significance of this case lies in the size and material of the foreign body and the extreme physical manipulation involved in urethral insertion by the patient. The successful use of a semi-rigid ureteroscope to navigate alongside the USB cable and achieve its removal under direct vision demonstrates the expansive nature of the urethra. Additionally, the complex nature of this patient’s inserted foreign body, including its overall thickness and material, shows the importance of undertaking a tailored approach to ensure minimally invasive removal and maintenance of urethral and bladder integrity.

## Conclusions

This report highlights multiple essential management principles in cases of emergency urethral foreign body extraction. While relatively rare, delayed presentation can lead to significant morbidities, including urethral trauma and infection. Prompt imaging and endoscopic evaluation are crucial for identifying both the location and characteristics of the foreign object. Our patient required careful endoscopic visualization and strategic cord removal to ensure a minimally invasive removal. This patient’s outcome highlights that this approach, even when presented with a larger and more complex foreign body, preserves the integrity of the urinary tract and minimizes complications. The presented patient notably had multiple previous instances of foreign body urethral insertions. Due to the high incidence of comorbid psychiatric disorders, a multidisciplinary approach with psychiatric evaluation should be undertaken to identify or exclude concomitant mental health conditions.
